# Prognostic Factors for Event-Free Survival in Pediatric Patients with Hepatoblastoma Based on the 2017 PRETEXT and CHIC-HS Systems

**DOI:** 10.3390/cancers11091387

**Published:** 2019-09-18

**Authors:** Hee Mang Yoon, Jisun Hwang, Kyung Won Kim, Jung-Man Namgoong, Dae Yeon Kim, Kyung-Nam Koh, Hyery Kim, Young Ah Cho

**Affiliations:** 1 Department of Radiology and Research Institute of Radiology, Asan Medical Center, University of Ulsan College of Medicine, Seoul 05505, Korea; espoirhm@gmail.com (H.M.Y.); youngbud27@paran.com (Y.A.C.); 2 Department of Radiology, Dongtan Sacred Heart Hospital, Hallym University Medical Center, Hwaseong 18450, Korea; biydjs@gmail.com; 3 Department of Pediatric Surgery, Asan Medical Center Children’s Hospital, University of Ulsan College of Medicine, Seoul 05505, Korea; namgoong2940@gmail.com (J.-M.N.); kimdy@amc.seoul.kr (D.Y.K.); 4 Division of Pediatric Hematology/Oncology, Department of Pediatrics, Asan Medical Center Children’s Hospital, University of Ulsan College of Medicine, Seoul 05505, Korea; pedkkn@amc.seoul.kr (K.-N.K.); taban@hanmail.net (H.K.)

**Keywords:** hepatoblastoma, pediatric, prognostic factor, PRETEXT, imaging

## Abstract

This study aimed to evaluate the prognostic value of variables used in the 2017 PRE-Treatment EXTent of tumor (PRETEXT) system and the Children’s Hepatic tumors International Collaboration-Hepatoblastoma Stratification (CHIC-HS) system in pediatric patients with hepatoblastoma. A retrospective analysis of data from the pediatric hepatoblastoma registry of a tertiary referral center was conducted to evaluate the clinical and imaging variables (annotation factors) of the PRETEXT staging system. The primary outcome was event-free survival (EFS). Data from 84 patients (mean age: 2.9 ± 3.5 years) identified between 1998 and 2017 were included. Univariable Cox proportional hazards analysis revealed that PRETEXT annotation factors P (portal vein involvement), F (multifocality of tumor), and M (distant metastasis) showed a significant negative association with EFS. Multivariable Cox proportional hazard analysis showed that factor F was the strongest predictor (HR (hazard ratio), 2.908; 95% CI (confidence interval), 1.061–7.972; *p* = 0.038), whereas factor M showed borderline significance (HR, 2.416; 95% CI, 0.918–6.354; *p* = 0.074). The prediction model based on F and M (F + M) showed good performance to predict EFS (C-statistic, 0.734; 95% CI, 0.612–0.854). In conclusion, the PRETEXT annotation factor F was the strongest predictor of EFS, and the F + M model showed good performance to predict EFS in pediatric patients with hepatoblastoma.

## 1. Introduction

Hepatoblastoma is the predominant hepatic malignancy in children [1]. Recent advances in diagnosis and treatment have improved patient outcomes and resulted in greater interest among healthcare professionals from a range of disciplines, particularly surgery, oncology, gastroenterology, and radiology. Indeed, advances in surgical treatment and chemotherapeutic combinations have dramatically increased the long-term survival rate to 75–80% [2]. Orthotropic liver transplantation has also broadened the range of patients that can be cured surgically [3].

Complete surgical resection of the hepatoblastoma is most likely to effect a cure, and pretreatment evaluation by imaging is crucial to define the extent of the disease, stratify risk, and plan treatment. Key treatment guidelines use the PRE-Treatment EXTent of tumor (PRETEXT) system as part of the risk stratification and treatment planning process. The original PRETEXT system for staging malignant primary liver tumors in children was designed by the International Childhood Liver Tumor Strategy Group (SIOPEL) in 1990. The prognostic value of the four PRETEXT categories has subsequently been demonstrated by the SIOPEL group [4,5], and PRETEXT has become the most widely used staging system for patients with pediatric hepatoblastoma. Additional criteria were added to the first revision of the PRETEXT system in 2005 [6], and the latest update (PRETEXT 2017) aimed to clarify definitions for its use in future collaborative trials [7]. The 2017 PRETEXT is part of the new international Hepatoblastoma Stratification (HS) system developed by the Children’s Hepatic tumors International Collaboration (CHIC) consortium; CHIC-HS was developed in response to the need to unify the disparate definitions and staging systems used by pediatric cooperative multicenter trial groups, thereby enabling the comparison of studies conducted by different groups in a variety of countries [8,9]. The new risk stratification system uses the following prognostic factors: PRETEXT group (I, II, III, or IV), age (<3, 3–7, and ≥8 years), alpha-fetoprotein (AFP) level ≤ 100 ng/mL, presence of metastasis, and PRETEXT annotation factors. 

Although the 2017 PRETEXT and CHIC-HS risk stratification systems have been evaluated in the large CHIC database from multiple pre-existing clinical trials, they have not been evaluated in a real-world setting, warranting validation based on robust data [10]. In addition, the current CHIC-HS system may be difficult to follow in clinical practice as there are many variables in its complex tree system. It is possible that identifying a limited number of strong predictors would be helpful in clinical practice to predict the prognosis of patients. Therefore, this study aimed to evaluate the prognostic value of each variable used in the 2017 PRETEXT and CHIC-HS systems, and to identify strong predictors of survival outcome in pediatric patients with hepatoblastoma.

## 2. Materials and Methods

### 2.1. Patients

A systematic, computerized search of a tertiary referral center database was retrospectively performed to identify potentially eligible patients between March 1998 and December 2017. The inclusion criteria were as follows: (1) histopathological diagnosis of hepatoblastoma; (2) age < 18 years; (3) abdominal computed tomography (CT) or magnetic resonance imaging (MRI) performed prior to treatment and initial imaging data available for analysis; and (4) available electronic medical records, including laboratory and follow-up data. 

The study was approved by the institutional review board of Asan Medical Center (No. 2017-1421), and the requirement for informed consent was waived due to the retrospective design. 

### 2.2. PRETEXT Staging System

In accordance with the recently updated 2017 PRETEXT staging system (Table S1) [7], the PRETEXT group and annotation factors were evaluated by two experienced pediatric radiologists (H.M.Y. and Y.A.C., with 5 and 19 years of experience in pediatric body imaging, respectively) by consensus, based on pretreatment CT or MRI images. The PRETEXT group indicates the overall tumor extent and depends on the number of hepatic sections that are free from tumor as follows: PRETEXT I, three contiguous hepatic sections free from tumor; PRETEXT II, two contiguous sections free from tumor; PRETEXT III, one section free from tumor; and PRETEXT IV, no tumor-free sections. Annotation factors include vascular involvement (V, hepatic vein/inferior vena cava; P, portal vein), extrahepatic tumor extension (E), multifocality (F), tumor rupture (R), caudate lobe involvement (C), lymph node metastases (N), and distant metastases (M). Subcategorization of hepatic and portal venous involvements was done based on the 2017 PRETEXT staging system [7] as follows: obliteration—tumor compressing the vein and no longer visible lumen; encasement—tumor touching and surrounding the vein by more than 50% or 180°; tumor thrombus—any thrombus within a first-order hepatic vein or the inferior vena cava for annotation V, and a first-order portal vein or the main portal vein for annotation P. All abdominal CT scans were performed with institutional contrast enhancement protocols including portal venous phase. In all patients, chest CT scans were performed to evaluate possible lung metastases. To determine the annotation factor M, all available imaging studies (positron emission tomography/CT scans, whole body MRIs, or bone scans) were thoroughly reviewed, if available. Surgical findings were also reviewed to check for possible misinterpretation of imaging studies, such as diaphragm involvement or peritoneal seeding. In addition, an aggregate factor of VPEFR, defined as the presence of at least one of V, P, E, F, or R factors, was assessed as described previously [10].

### 2.3. Clinical Data Collection for CHIC-HS

The study focused on clinical characteristics identified as significant risk factors in previous reports of the CHIC-HS risk stratification of pediatric hepatoblastoma [9,10]. In accordance with the CHIC-HS [9,10], patient age was classified into three groups (≤2, 3–7, and ≥8 years) and serum AFP concentration into four groups (<100, 100–999, 1000–10^6^, and >10^6^ ng/mL). 

### 2.4. Statistical Analysis 

The primary outcome of this study was event-free survival (EFS), which was defined as time from enrollment (the date of an initial abdominal CT scan) until the occurrence of an event: first relapse, disease progression, development of second malignancy, or death for any reason [10]. Relapse and disease progression were evaluated using the Response Evaluation Criteria in Solid Tumors (RECIST) 1.1 criteria [11]. The last follow-up date was recorded. To evaluate the prognostic value of each variable, the cumulative event-free survival curves were analyzed according to the Kaplan–Meier (KM) method and the log-rank test was performed to compare the survival rates between patients with variable and without variable. To identify strong predictors of EFS, univariate and multivariate Cox regression analysis were performed. Variables showing a tendency toward statistical significance (*p* < 0.1) in the univariable model were entered into the multivariable model. Variables were selected in a backward elimination manner. Variable risk was expressed as a hazard ratio (HR) with a corresponding 95% confidence interval (CI). C-statistics with a corresponding 95% CI were calculated based on the multivariate Cox regression model. C-statistics were regarded as follows: >0.8 indicates excellent discrimination, 0.7–0.8 indicates good discrimination, 0.6–0.7 indicates some clinical value, and <0.6 indicates no clinical value [12]. For model validation, internal validation was performed using a bootstrapping method with 1000 resamples. Bootstrapping yields an optimism-corrected C-statistic which is calculated by subtracting optimism from the C-statistic in the original cohort according to Harrell’s algorithm [13].

The KM curves for recurrence-free survival (RFS) rate and overall survival (OS) rate were also generated to summarize survival probabilities, and the low-rank test was used to test the prognostic significance of age, AFP, PRETEXT groups, and PRETEXT annotation factors. The RFS was defined as the period from the date of diagnosis to that of first relapse. The OS was defined from the date of diagnosis to that of death, the last confirmation of the patient being alive, or the most recent follow-up.

Quantitative variables were presented using descriptive statistics, such as the mean ± standard deviation (SD). A *p*-value < 0.05 was considered to be indicative of a statistically significant result. Statistical analysis was conducted using SPSS (version 21; SPSS, Chicago, IL, USA) and R software version 3.4.4 [14]. 

## 3. Results

### 3.1. Baseline Patient Characteristics

A total of 125 potentially eligible patients were identified. Among these, 41 patients were excluded (21 were diagnosed with other diseases, 14 had no initial CT or MRI data, and 6 had no available follow-up medical records). The baseline characteristics of the 84 eligible patients are summarized in Table 1. Type of treatment and clinical course are summarized in Table 1 and Supplementary Figure S1. The mean age of patients was 2.9 ± 3.5 years, and 56 patients (66.7%) were ≤2 years of age. The median follow-up was 4.7 years (range, 0.01–19.70 years) for patients without an event. Serum AFP concentration was missing in two patients who had been referred from another hospital. Most patients (79/84) had serum AFP concentrations >1000 ng/mL. One patient with an AFP level <100 ng/mL and two patients whose AFP levels were >100 but <1000 ng/mL were assessed as a single subgroup due to the insufficient number of patients for the statistical analysis of individual groups. Of the 84 patients, 23 (27.4%) presented with distant metastasis at initial diagnosis. Lung metastases were found in all 23 patients and one had synchronous multiple bone metastases. Surgical treatment of patients with annotation factor V and P-positive is summarized in Supplementary Table S2. Histologic subtype was available for 77 patients. The tumors were classified as either epithelial (*n* = 57) or mixed epithelial and mesenchymal (*n* = 20). The epithelial type was further classified as predominantly fetal epithelial (*n* = 29), mixed epithelial (including predominantly embryonal and mixed fetal and embryonal) (*n* = 16), macrotrabecular (*n* = 4), small cell undifferentiated (*n* = 3), and not classified (*n* = 5). The effect of histologic subtype on EFS is presented in Supplementary Figure S2.

### 3.2. Prognostic Values of Each Variable Used in the 2017 PRETEXT and CHIC-HS Systems

**Figure 1:**
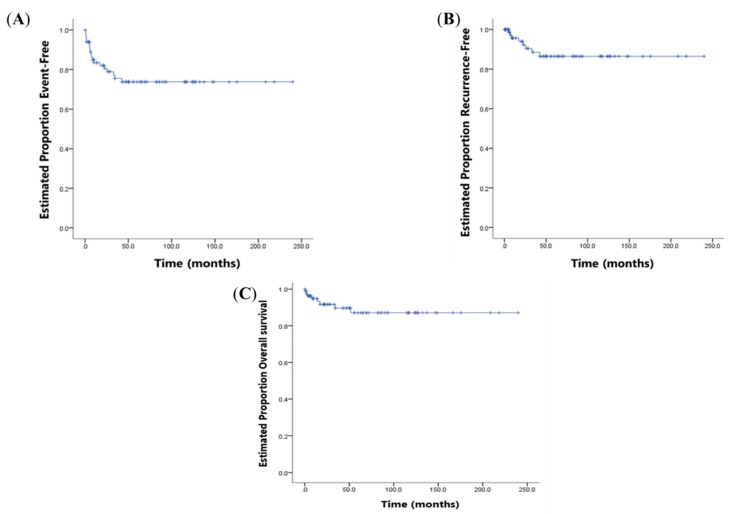
Overall cumulative survival curves for event-free survival (**A**), recurrence-free survival (**B**), and overall survival (**C**) in 84 patients with hepatoblastoma.

A total of 19 events to calculate EFS were observed: eight patients relapsed, nine had disease progression, and two died. None of the patients had secondary malignancies. The mean EFS was 180.6 months (95% CI, 157.5–203.8 months; Figure 1A).

**Figure 2:**
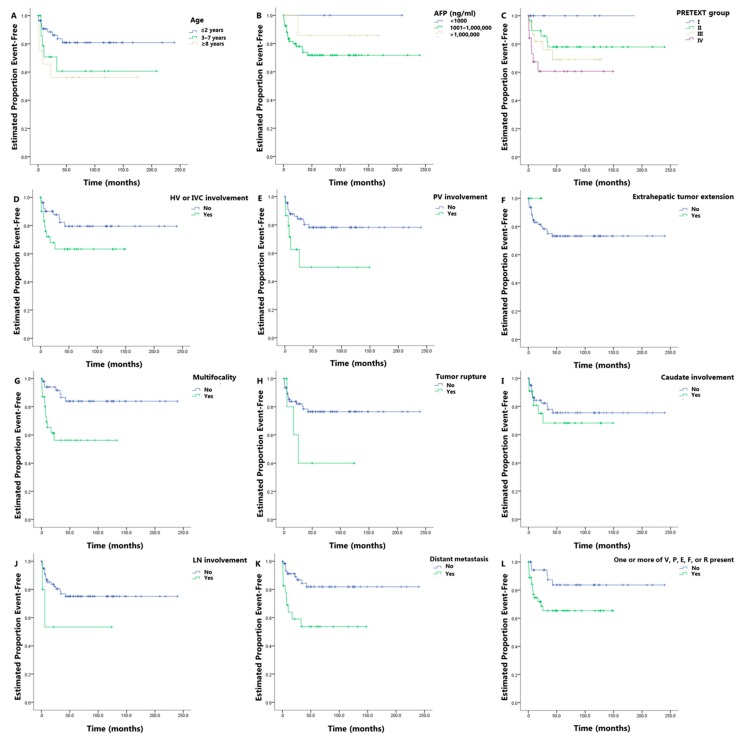
Kaplan–Meier curves of event-free survival (EFS) according to age (**A**), serum alpha-fetoprotein (AFP) levels at initial presentation (**B**), PRETEXT group (**C**), HV or IVC involvement (**D**), portal vein involvement (**E**), extrahepatic tumor extension (**F**), multifocality (**G**), tumor rupture (**H**), caudate involvement (**I**), lymph node involvement (**J**), distant metastasis (**K**), and presence of one or more of VPEFR (**L**). The detailed information for each graph is described in the supplementary figure legend.

The KM plots for EFS in variables including age, AFP, PRETEXT group, and PRETEXT annotation factor are shown in Figure 2. The mean EFS and three- and five-year EFS rate associated with these variables are summarized in Table 2. The survival curves in all these variables showed differences between the stratified subsets of patients, suggesting prognostic value; however, not all variables showed statistical significance. The PRETEXT annotation factors P (negative vs. positive, *p* = 0.030), F (negative vs. positive, *p* = 0.002), M (negative vs. positive, *p* = 0.003), and VPEFR (negative vs. positive, *p* = 0.035) showed a significant association with EFS in the log-rank test. Borderline significance was noted in the age groups (≤2 years vs. 3–7 years vs. ≥8 years, *p* = 0.078) and PRETEXT annotation factor V (negative vs. positive, *p* = 0.079).

Overall cumulative survival curves for RFS and OS are presented in Figure 1B,C. The mean RFS was 210.3 months (95% CI, 191.1–229.4 months) and the mean OS was 211.6 months (95% CI, 192.9–230.3 months). The KM plots for RFS and OS comparing patients with variables and without variables (i.e., age, AFP, PRETEXT group, and PRETEXT annotation factor) are present in Supplementary Figures S3 and S4, respectively. The annotation factor R (negative vs. positive, *p* = 0.020) was associated with a significantly worse RFS. The PRETEXT annotation factors P (negative vs. positive, *p* < 0.001), F (negative vs. positive, *p* = 0.006), and N (negative vs. positive, *p* = 0.003) showed a significant association with OS.

### 3.3. Prognostic Factors for Predicting Event-Free Survival

In the univariate Cox proportional hazards model, PRETEXT annotation factors P, F, and M, and the aggregation factor VPEFR at the time of initial diagnosis, showed a significant association with EFS (Table 3). The prediction model based on the PRETEXT group (C-statistic, 0.663; 95% CI, 0.535–0.791) and aggregation factor of VPEFR (C-statistic, 0.639; 95% CI, 0.523–0.755) showed tendency of prognostic value to predict EFS (Supplementary Table S3).

Multivariable Cox proportional hazard analysis showed that PRETEXT annotation factor F was the strongest predictor (HR, 2.908; 95% CI, 1.061–7.972; *p* = 0.038). PRETEXT annotation factor M showed a borderline significance in predicting EFS (HR, 2.416; 95% CI, 0.918–6.354; *p* = 0.074). The prediction model based on F and M (F + M model), derived from the multivariate Cox proportional hazards model, showed good performance to predict EFS, with a C-statistic of 0.734 (5% CI, 0.612–0.854). Bootstrapping method yielded an optimism-corrected C-statistic of 0.718 (95% CI, 0.598–0.837). The similarity between the C-statistic and optimism-corrected C-statistic might suggest robustness of this prediction model (Table S3).

## 4. Discussion

This study analyzed the prognostic value of each variable used in the 2017 PRETEXT staging system and the CHIC-HS system and evaluated their association with EFS. With the exception of serum AFP concentration and PRETEXT annotation factor E, all variables showed a tendency to indicate increased risk, which is in agreement with the results of previous studies [9,10]. However, a statistically significant association was observed only with PRETEXT annotation factors P, F, and M, and one or more of VPEFR in the univariate analysis. Multivariate analysis showed that PRETEXT annotation factor F was the strongest negative prognostic factor and M showed borderline significance. The prediction model combining F and M showed good discrimination in our cohort.

The international risk stratification system recently developed by the CHIC consortium was formed in response to the need to unite data from four multicenter trial groups: SIOPEL, the Children’s Oncology Group; the German Society for Pediatric Oncology and Hematology; and the Japanese Study Group for Pediatric Liver Tumors [8,9]. In addition to the traditional poor prognostic indicators, such as a low AFP level [15,16], advanced PRETEXT group [4,15,16], older age [16,17,18,19], and distant metastases [16,20], this large CHIC database found that PRETEXT annotation factors V, P, E, F, and R, and the aggregate of VPEFR, were significantly associated with an increased risk of EFS events [9].

In the current study, with the exception of serum AFP level and PRETEXT annotation factor E, hazard ratios of all variables used in the 2017 PRETEXT and CHIC-HS systems pointed in the same direction as those in previous studies, although not all variables were statistically significant [9,10]. Among variables derived from the 2017 PRETEXT, we found that annotation factors P, F, and M, and one or more of VPEFR were significant prognostic factors, which is in agreement with previous studies. However, PRETEXT annotation factor V showed only borderline significance. These results suggest that extensive hepatic vascular involvement, extensive intrahepatic parenchymal involvement, and extrahepatic metastasis may be associated with poor clinical outcomes. In addition, hepatic vascular involvement (V, P) may be linked with multifocal intrahepatic (F) and extrahepatic tumor metastasis (M). Among the aggregation factors VPEFR, E and R were not seen to be significant prognostic indicators in this study. However, these observations are limited by the small number of patients (four and seven patients being E- and R-positive, respectively); the role of intra-abdominal extrahepatic tumor spread (E and R) therefore requires validation in a larger patient cohort.

The CHIC-HS is primarily based on the PRETEXT group, which represents the extent of the liver to be resected [9]; multiple previous trials have confirmed that the PRETEXT group is a powerful predictor of overall survival in children with hepatoblastoma [21]. Our study showed prognostic values of PRETEXT groups, as illustrated in Figure 2C which is the Kaplan–Meier curve showing that the prognosis worsens as the number of PRETEXT group increases. However, these prognostic values did not reach statistical significance on the log-rank test and univariable Cox proportional hazard model probably due to the small number of outcome events (*n* = 19). Although the C-statistic of the univariable Cox proportional hazard model based on PRETEXT groups was 0.663, we could not add the PRETEXT groups in the multivariable Cox proportional hazard model. As complete surgical excision is the cornerstone of treatment for hepatoblastoma, it will be important to re-evaluate the prognostic role of the PRETEXT groups in a larger cohort of patients [10].

Among the clinical variables at initial diagnosis of age and serum AFP level derived from the CHICH-HS system, only age showed borderline significance as a prognostic factor. In previous studies, the prognostic role of older age has been inconsistent [5,16]. The CHIC study, which included a large population and was therefore statistically robust, found older age to be associated with negative outcomes [9]. A previous study has also reported that patients with an earlier age of onset expressed chemosensitive membranous molecules and β-catenin [22]. Another study found that a transitional liver cell tumor, although it is a rare subtype of hepatoblastoma, occurred in older children and young adults and was associated with a poor prognosis [23]. In the current study, no significant differences in prognosis were seen between the serum AFP level subgroups, although a very low serum AFP level is well established as a poor prognostic factor in patients with hepatoblastoma [4,15,16,20]. In previous studies, very low serum AFP levels (<100 ng/mL) and borderline low AFP levels (100–999 ng/mL) were associated with poor outcomes, while very high serum levels (>10^6^ ng/mL) were also associated with elevated risk [9,10]. In the current study, no patients had an EFS event in the very low or borderline low AFP level groups, although this observation is limited by the small number of patients in these subgroups (AFP <100 ng/mL, *n* = 1; AFP 100–999 ng/mL, *n* = 2). In addition, although the results did not achieve statistical significance, patients in the higher AFP level subgroup (>10^6^) tended to show better EFS outcomes, which is not in line with reports from previous studies [9,16]. Another previous study of Asian patients included only a single patient with very low serum AFP levels and did not report their outcome [24]. The small patient numbers to date mean that further evaluation will be required to establish whether there is any association between genetic and environmental factors that contribute to the clinical outcome of serum AFP levels in different subsets of Asian patients with hepatoblastoma. Further evaluation of the prognostic effect of higher AFP levels (>10^6^) is also recommended.

PRETEXT annotation factor F was found to be the most powerful prognostic indicator in the current study. The CHIC and the SIOPEL I, II, and III studies have also reported the negative prognostic effect of multifocality, as also seen in the current study [5,16]. According to a previous study, which used the SIOPEL protocol for treatment, multifocality was independently associated with worse EFS (HR, 10.1) [25]. The authors suggest that patients with multifocal tumors should undergo intensive chemotherapy. Although the study was limited by small patient numbers, these results, and those of previous studies, highlight the fact that microscopic residual tumor tissue in livers that appear to be clear on imaging might lead to relapse after resection of the multifocal tumor alone [26,27]. PRETEXT annotation factor M was a significant indicator of reduced EFS in our study, although it showed only borderline significance on multivariate analysis. The current study identified distant metastasis in 27.4% of patients, primarily to the lungs. This level of prevalence is slightly higher than that reported in previous studies (17–18%), which have confirmed the negative influence of metastasis on patient outcomes [4,9,16]. The F + M model derived from the multivariate analysis in the current study showed good discrimination, with a C-index of 0.734 for predicting EFS. This model consists of imaging factors but not clinical factors and, therefore, pretreatment imaging studies are of significant value when predicting outcomes in patients with hepatoblastoma. As the PRETEXT annotation factor F was the most powerful predictor, radiologists should carefully evaluate multifocal tumor spread in the liver. When CT cannot provide adequate image quality to evaluate multifocal tumors, other imaging techniques, such as ultrasound or MRI, should be considered as alternatives. MRI provides better soft tissue contrast than CT in liver imaging, and the availability of a liver-specific contrast media has also improved the detection and characterization of lesions [28,29]. CT is currently the main imaging modality used in the pretreatment liver evaluation of patients with hepatoblastoma, and further studies are required to determine the value of liver MRI in the detection of multifocality. In addition, meticulous pretreatment imaging is required to evaluate distant metastasis, particularly in the lungs. It may not be possible to generalize the results seen in the current single-center study, particularly as the data are limited by the small number of patients. The F + M model should, therefore, be validated as part of a large-scale, multicenter study.

Annotation factors C and N, which were not included in the previous CHIC study [9], showed no prognostic significance in our study. In our study, the involvement of the caudate lobe could impact on increasing surgical resection extent such as extended right lobectomy or extended left lobectomy, however such a change might not impact EFS [7]. Lymph node metastasis is rare in patients with hepatoblastoma, even though one study reported that hilar lymph node enlargement is associated with reduced EFS [5]. We postulate that the rarity of lymph node metastasis might limit the establishment prognostic significance.

Regarding the effect of histologic subtypes on EFS, the macrotrabecular and small cell undifferentiated subtypes showed worse prognosis than other subtypes. Our results are in line with previous studies that have reported better prognosis of pure fetal well-differentiated type and worse prognosis of macrotrabecular and small cell undifferentiated types [30,31]. However, we did not include histologic subtypes in our univariable and multivariable cox proportional hazards model, because the 2017 PRETEXT and CHIC-HS systems do not contain histologic subtypes.

The current study has several limitations. First, as hepatoblastoma is a rare disease, our study included a relatively small number of patients (*n* = 84), which is a similar cohort size to the previous study conducted by the major cooperative trial group of the CHIC database (*n* = 72) [32]. Better investigation of prognostic predictors in this rare type of pediatric tumor would be achieved by the future retrospective and prospective validation process as discussed by Meyers et al. [9,10]. Second, although the F + M model derived from our study showed good discrimination, it is limited by the fact that the PRETEXT group was not considered. The F + M model therefore requires further validation in additional studies together with CHIC-HS. Third, our study did not analyze the effect of molecular variables on EFS. Some research has been conducted to investigate the molecular biology and prognostic factors associated with hepatoblastoma [33], and there may be some interplay between clinico-radiologic predictors and molecular biology. However, mutation status analysis is not available in daily practice, and future studies should incorporate molecular variables into staging systems when evaluating novel targeted therapies.

Despite these limitations, our study provides supporting evidence of utility of the 2017 PRETEXT and CHIC-HS system by providing good prognostic value of its variables. Our study is also meaningful to evaluate the external generalizability of the 2017 PRETEXT and CHIC-HS system based on real-world data from our geographically different cohort.

## 5. Conclusions

In this cohort of pediatric patients with hepatoblastoma, PRETEXT annotation factors P, F, and M and the aggregate of VPEFR showed a significant association with patient outcome, with factor F being the strongest predictor of EFS among the variables used in the 2017 PRETEXT and CHIC-HS systems. The F + M model derived from this study showed good performance in predicting EFS in this patient group.

## Figures and Tables

**Table 1:** Baseline patient and tumor characteristics.

Characteristic	Category	N (%) or Mean ± Standard Deviation
Total number of patients		84
Age at initial diagnosis (years)		2.9 ± 3.5
	≤2	56 (66.7)
	3–7	12 (14.3)
	≥8	9 (10.7)
Sex (male:female)		48:36
Serum AFP concentration, ng/mL	<100	1 (1.2)
	100–999	2 (2.4)
	1000–10^6^	69 (82.1)
	>10^6^	10 (11.9)
	missing	2 (2.4)
PRETEXT group	I	12 (14.3)
	II	29 (34.5)
	III	23 (27.3)
	IV	20 (23.8)
Annotation factors		
V (HV or IVC involvement)	Yes	31 (36.9)
Tumor obliterates or encases all three HV	Yes	9 (10.7)
Tumor obliterates or encases IVC	Yes	21 (25)
Tumor thrombus in HV or IVC	Yes	7 (8.3)
P (PV involvement)	Yes	15 (17.9)
Tumor obliterates or encases both PV	Yes	6 (7.1)
Tumor obliterates or encases main PV	Yes	5 (6.0)
Thrombus within a first-order PV or main PV	Yes	8 (9.5)
E (extrahepatic tumor extension)	Yes	4 (4.8)
F (multifocality)	Yes	32 (38.1)
R (tumor rupture)	Yes	7 (8.3)
C (caudate involvement)	Yes	22 (26.2)
N (lymph node metastasis)	Yes	5 (6.0)
M (distant metastasis)	Yes	23 (27.4)
One or more of V, P, E, F, or R	Yes	46 (54.8)
Number of patients with an event		19 (22.6)
Number of deaths		8 (9.5)
Preoperative chemotherapy		81 (96.4)
Cisplatin/doxorubicin		5 (5.9)
Cisplatin/5-fluorouracil/vincristine		46 (54.7)
Cisplatin/5-fluorouracil/vincristine/doxorubicin		22 (26.1)
Ifosfamide/carboplatin/etoposide		4 (4.7)
Missing		4 (4.7)
Surgical resection		54 (64.2)
Orthotopic liver transplantation		17 (20.2)
Resection of pulmonary metastases		13 (15.4)

Abbreviations: AFP—alpha-fetoprotein; PRETEXT—2017 PRE-Treatment EXTent of tumor; HV—hepatic vein; IVC—inferior vena cava; PV—portal vein.

**Table 2:** The mean event-free survival (EFS) and three- and five-year EFS rates according to each variable used in the 2017 PRE-Treatment EXTent of tumor (PRETEXT) and Children’s Hepatic tumors International Collaboration-Hepatoblastoma (CHIC-HS) systems.

Variables		EFS, Months		3 Year EFS Rate, %	5 Year EFS Rate, %	*p*-Value
		Mean	95% CI			
Overall		180.6	157.5–203.8	75.6	73.8	
Age at initial diagnosis (years)	≤2	197.3	172.0–222.6	83.6	80.9	**0.078**
	3–7	131.9	78.4–185.5	60.6	60.6	
	≥8 years	102.0	53.3–150.8	56.3	56.3	
AFP, ng/mL	<1000	*	*	100	100	0.389
	1000–10^6^	175.9	149.8–202.0	73.9	71.7	
	>10^6^	146.3	109.8–182.8	85.7	85.7	
PRETEXT group	I	*	*	100	100	0.106
	II	190.7	155.9–222.5	77.9	77.9	
	III	93.7	70.7–116.6	75.9	69.0	
	IV	92.7	60.0–125.5	60.6	60.6	
Annotation factors						
V (HV or IVC involvement)	No	194.6	167.9–221.2	82.3	79.5	**0.079**
	Yes	97.6	72.0–123.3	63.3	63.3	
P (PV involvement)	No	191.0	167.4–214.7	80.3	78.2	**0.030**
	Yes	79.9	38.5–121.3	50.1	50.1	
E (extrahepatic tumor extension)	No	179.3	155.8–202.8	75.0	73.2	0.456
	Yes	*	*	**	**	
F (multifocality)	No	204.9	181.1–228.7	86.5	83.9	**0.002**
	Yes	78.1	54.4–101.7	56.2	56.2	
R (tumor rupture)	No	186.6	163.5–209.7	78.4	76.5	0.089
	Yes	59.2	12.5–105.9	40.0	40.0	
C (caudate involvement)	No	184.9	158.6–211.1	77.9	75.5	0.492
	Yes	105.2	75.7–134.6	68.2	68.2	
N (lymph node metastasis)	No	183.8	160.5–207.1	76.9	75.1	0.133
	Yes	67.9	9.9–125.8	53.3	53.3	
M (distant metastasis)	No	199.7	175.8–223.7	84.3	81.9	**0.003**
	Yes	83.2	53.8–112.7	53.6	53.6	
One or more of V, P, E, F, or R present	No	204.8	176.6–233.0	87.3	83.7	**0.035**
	Yes	100.6	79.7–121.4	65.4	65.4	

* The mean EFS of patients was not estimated because no event was observed in this group. ** Three- and five-year EFS rates were not estimated because data were censored before three years in this group. Abbreviations: CI—confidence interval; AFP—alpha-fetoprotein; HV—hepatic vein; IVC—inferior vena cava; PV—portal vein. *p*-Values outlined in bold demarcate either a statistical significance or borderline significance.

**Table 3:** Cox proportional hazards model with variables used in the 2017 PRETEXT and CHIC-HS systems.

Variables	Univariate			Multivariate		
	Hazard Ratio	95% CI	*p*-Value	Hazard Ratio	95% CI	*p*-Value
Age at initial diagnosis (years)						
≤2	1		0.094			
3–7	2.352	0.787–7.034	0.648			
≥8	3.035	1.016–9.064				
AFP, ng/mL						
<1000	Infinite		0.991			
1000–10^6^	1		0.568			
>10^6^	0.390	0.052–2.933				
PRETEXT group						
I	Infinite		0.991			
II	1		0.444			
III	1.384	0.445–4.302				
IV	2.456	0.820–7.355				
Annotation factors						
V (HV or IVC involvement)	2.205	0.895–5.435	0.086			
P (PV involvement)	2.819	1.062–7.483	**0.037**			
E (extrahepatic tumor extension)	Infinite		0.993			
F (multifocality)	3.959	1.564–10.135	**0.004**	2.908	1.061–7.972	**0.038**
R (tumor rupture)	2.789	0.811–9.597	0.104			
C (caudate involvement)	1.400	0.532–3.688	0.495			
N (lymph node metastasis)	2.939	0.671–12.875	0.153			
M (distant metastasis)	3.566	1.448–8.786	**0.006**	2.416	0.918–6.354	0.074
One or more of V, P, E, F, or R present	2.879	1.034–8.018	**0.043**			

Abbreviations: AFP—alpha-fetoprotein; HV—hepatic vein; IVC—inferior vena cava; PV—portal vein. *p*-Values outlined in bold demarcate a statistical significance.

## Supplementary Materials

The following are available online at https://www.mdpi.com/2072-6694/11/9/1387/s1, Table S1: 2017 PRETEXT groups and annotation factors, Table S2. Surgical treatment of patients with annotation factors V and P-positive, supplementary figure legend for Figure 2, Figure S1: Clinical course of patients, Figure S2: Effect of histologic subtypes on event-free survival, Figure S3: Kaplan-Meier (KM) curves of recurrence-free survival (RFS), Figure S4.: Kaplan-Meier (KM) curves of overall survival (OS).
