# Supplementary material for: Prognostic Factors for Event-Free Survival in Pediatric Patients with Hepatoblastoma Based on the 2017 PRETEXT and CHIC-HS Systems
Source: Cancers (Basel). 2019 Sep 18;11(9):1387. doi: 10.3390/cancers11091387 (PMC6769992; doi:10.3390/cancers11091387)
Supplement: Supplementary file 1 [file cancers-11-01387-s001.pdf]

Supplementary Materials: Prognostic Factors for Event-Free Survival in Pediatric Patients with Hepatoblastoma Based on The 2017 PRETEXT and CHIC-HS Systems

Hee Mang Yoon, Jisun Hwang, Kyung Won Kim, Jung-Man Namgoong, Dae Yeon Kim, Kyung-Nam Koh, Hyery Kim, and Young Ah Cho

Table S1. 2017 PRETEXT groups and annotation factors.

PRETEXT Groups	Definition
I	3 contiguous hepatic sections are free of tumor
II	2 contiguous hepatic sections are free of tumor
III	1 contiguous hepatic section is free of tumor
IV	Tumor affects all four hepatic sections (almost always multifocal or infiltrative)
**Annotation Factors**	**Positive Definition**
Hepatic venous/IVC involvement (V)	Any of the following criteria:<br>Tumor obliterates or encases all three hepatic veins[a] or the intrahepatic IVC<br>Tumor thrombus in any hepatic vein[a] or the intrahepatic IVC
Portal venous involvement (P)	Any of the following criteria:<br>Tumor obliterates or encases either both portal veins[b] or the main portal vein<br>Tumor thrombus in either or both the right and left portal veins[b] or the main portal vein
Extrahepatic tumor extension (E)	Any of the following criteria:<br>Tumor crosses boundaries or tissue planes<br>Tumor is surrounded by normal tissue more than 180°<br>Peritoneal nodules: one nodule ≥ 10 mm or two or more nodules ≥ 5 mm
Multifocality (F)	Two or more separate hepatic tumors with normal liver tissue between the tumors
Tumor rupture (R)	Any findings of hemorrhage on imaging (Ultrasound, CT, MRI)<br>Pathologically diagnosed after paracentesis/upfront resection
Caudate lobe (C)	Tumor involving the caudate lobe
Lymph node metastasis (N)	Considered when any of the following morphologic criteria is met (less sensitive for detection):<br>Lymph node with short-axis diameter > 1 cm<br>Portocaval lymph node with short-axis diameter > 1.5 cm<br>Spherical lymph node shape with absent fatty hilum
Distant metastasis (M)	Any of the following criteria for pulmonary metastasis:<br>Single non-calcified lung lesion with diameter ≥ 5 mm<br>Two or more non-calcified lung lesions with each diameter ≥ 3 mm<br>Any metastases diagnosed via pathology

IVC = inferior vena cava. [a] First-order hepatic vein: between its confluence with IVC and its most central branch. [b] First-order portal vein: between the bifurcation and the first major branch of the portal vein.

Table S2. Surgical treatment of patients with annotation factors V and P-positive.

Criteria for the assignment of V and P-positive	Total	Resection, N (%)	OLTx, N (%)
V (HV or IVC involvement)	31		
Tumor obliterates or encases all three HV	9	1 (11.1)	4 (44.4)
Tumor obliterates or encases IVC	21	7 (33.3)	5 (23.8)
Tumor thrombus in HV	3	2 (66.7)	1 (33.3)
Tumor thrombus in IVC	4	3 (75.0)	0
P (PV involvement)	15		
Tumor obliterates or encases both PV	6	2 (33.3)	2 (33.3)
Tumor obliterates or encases main PV	5	1 (20.0)	2 (40.0)
Thrombus within a first-order PV	7	0	1 (14.3)
Thrombus within main PV	1	0	1 (100)

OLTx, orthotopic liver transplantation; HV, hepatic vein; IVC, inferior vena cava; PV, portal vein.

Table 3. Performance measures of Cox proportional hazard models to predict EFS.

Model Type	Discrimination	
	**C-statistic**	**Optimism-Corrected C-Statistic***
PRETEXT group	0.663 (0.535–0.791)	0.623 (0.495–0.751)
VPEFR†	0.639 (0.523–0.755)	0.636 (0.520–0.752)
F+M model	0.734 (0.612–0.854)	0.718 (0.598–0.837)

Data in parentheses are 95% confidence intervals; * Optimism-corrected C-statistic obtained from bootstrapping method; † One or more of V, P, E, F, or R present.

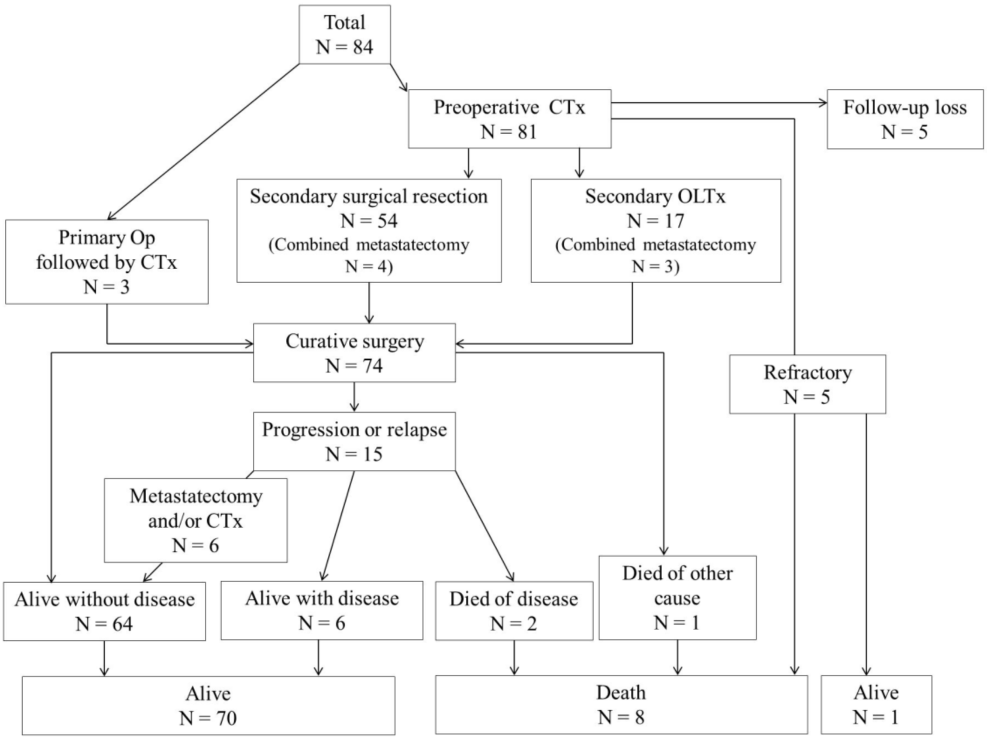

Figure S1. Clinical course of patients. CTx, chemotherapy; Op, operation; OLTx, orthotopic liver transplantation.

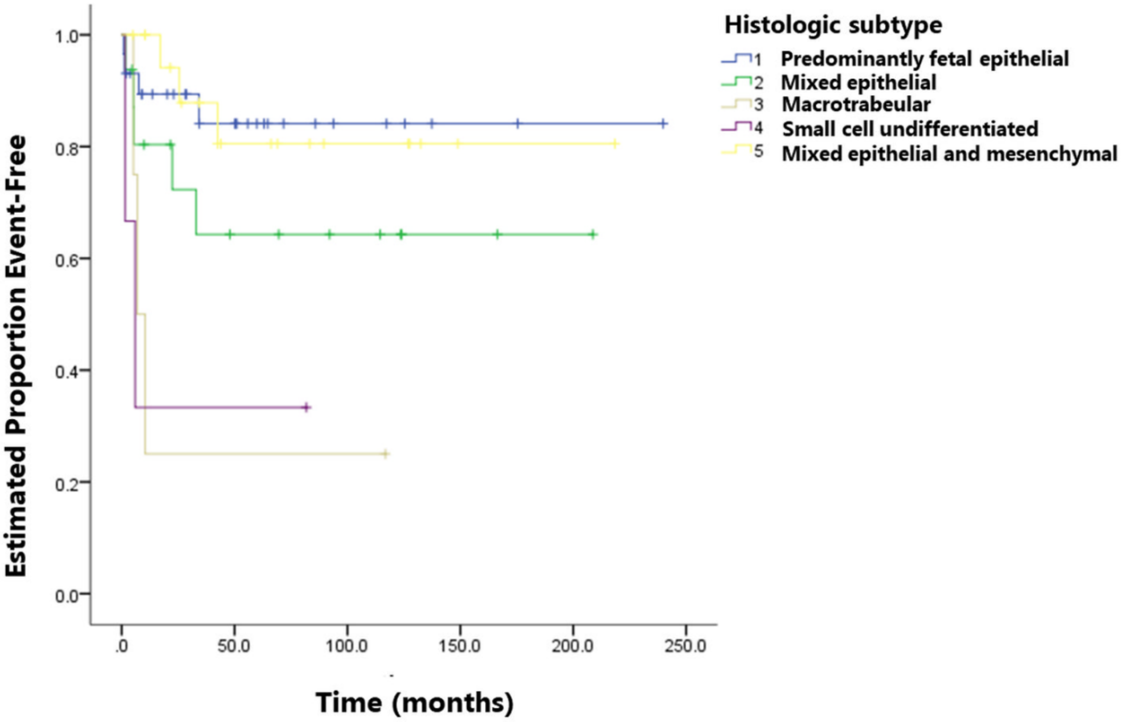

Figure S2. Effect of histologic subtypes on event-free survival. In the Kaplan–Meier curves of event-free survival according to the histologic subtype, the mean EFS was 203.9 months (95% CI of 171.1–236.6 months) for patients with predominantly fetal epithelial subtype, 139.4 months (95% CI of 90.1–188.7 months) for patients with mixed epithelial subtype, 34.9 months (95% CI of 0.0–81.2 months) for patients with macrotrabecular subtype, 29.8 months (95% CI of 0.0–71.5 months) for patients with small cell undifferentiated subtype, and 181.6 months (95% CI of 144.0–219.2 months) for patients with mixed epithelial and mesenchymal subtype, respectively. There were statistical significances in mean EFS between predominantly fetal epithelial subtype vs. macrotrabeular subtype (p = 0.004), and predominantly fetal epithelial subtype vs. small cell undifferentiated subtype (p = 0.017).

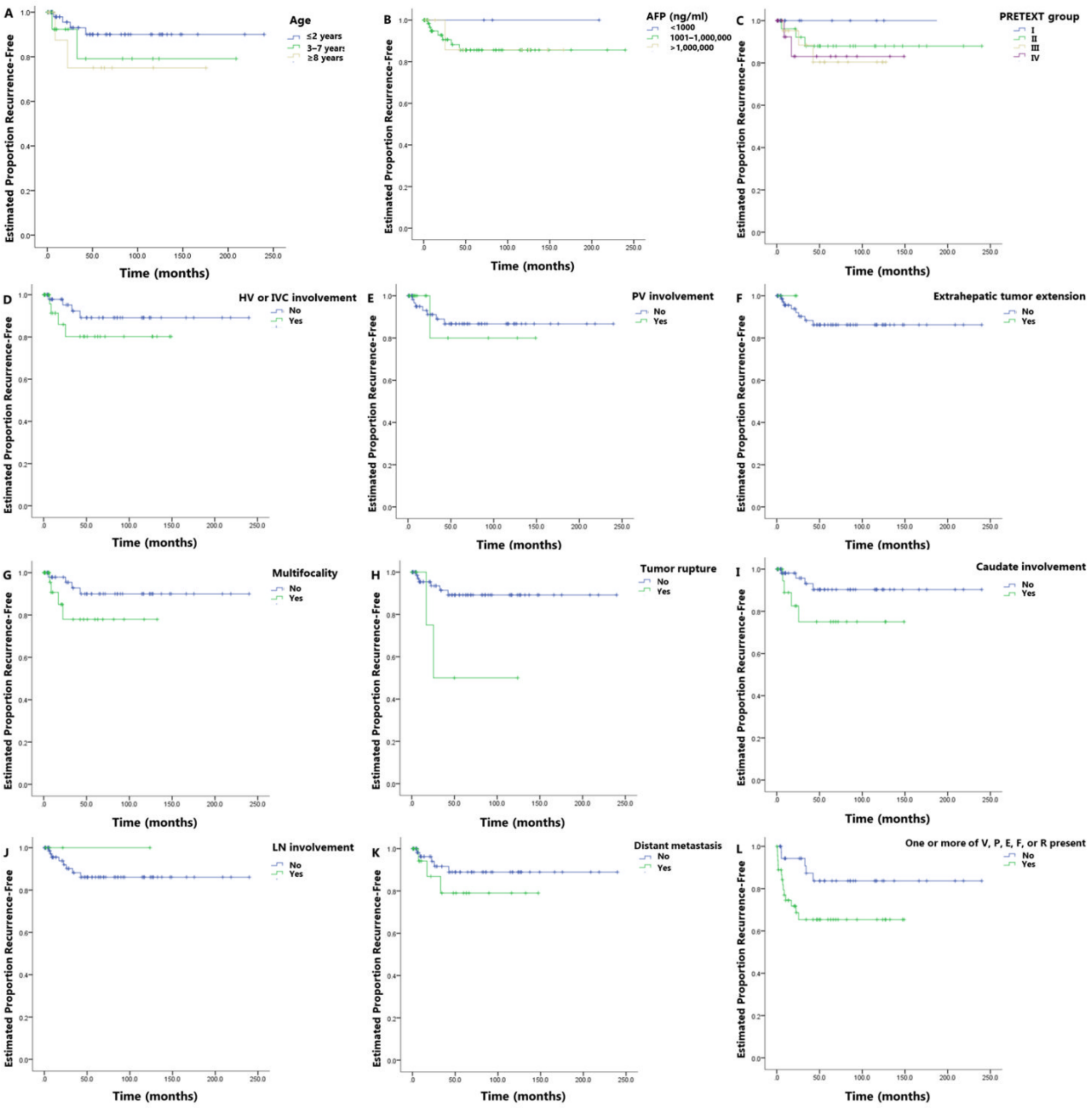

Figure S3. Kaplan-Meier (KM) curves of recurrence-free survival (RFS). Figure 3**A**. KM curves according to age at initial presentation. The mean RFS was 218.3 months (95% CI, 198.2−238.4 months) for patients ≤ 2 years, 169.9 months (95% CI of 120.5−219.2 months) for patients aged 3−7 years, and 135.5 months for patients ≥ 8 years (95% CI of 87.5−183.6 months), respectively. There was no significant difference in mean RFS among three groups (p = 0.376). Figure 3**B**. KM curves according to serum alpha-fetoprotein (AFP) levels at initial presentation. The mean RFS of patients with AFP < 1000nng/mL was not estimated because no event was observed in this group. The mean RFS was 208.4 months (95% CI, 186.6–230.1 months) for patients with AFP of 1001–1000000ng/mL, and 146.3 months (95% CI, 109.8–182.8 months) for patients with AFP more than 1000000 ng/m, respectively. There was no significant difference in mean RFS between two groups (p = 0.938). Figure 3**C**. KM curves according to PRETEXT group. The mean RFS was 213.4 months (95% CI, 185.4–241.5 months) for patients with group II, 108.1 months (95% CI, 88.0–128.2 months) for patients with group III, and 125.9 months (95% CI, 96.8–155.1 months) for patients with group IV, respectively. The mean RFS of PRETEXT group I was not estimated because no event had been observed in this group. There was no significant difference in mean RFS among four groups (p = 0.657). Figure 3**D**. KM curves according to HV or IVC involvement (V). The mean RFS was 216.7 months (95% CI, 195.2–238.2 months) for patients with negative V, and 122.5 months (95% CI, 99.1–145.9 months) for patients with positive V, respectively. There was no significant difference in mean EFS between two groups (p = 0.271). Figure 3**E**. KM curves according to PV involvement (P). The mean RFS was 210.7 months (95% CI, 190.5–230.9 months) for patients with negative P, and 124.2 months (95% CI, 81.0–167.5 months)

for patients with positive P, respectively. There was no significant difference in mean RFS between two groups (p = 0.950). Figure 3**F**. KM curves according to extrahepatic tumor extension (E). The mean RFS was 209.8 months (95% CI, 190.3–229.2 months) for patients with negative E. The mean EFS of patients with positive E was not estimated because no event had been observed in this group. There was no significant difference in mean EFS between two groups (p = 0.703). Figure 3**G**. KM curves according to multifocality (F). The mean RFS was 218.5 months (95% CI, 198.7–238.4 months) for patients with negative F, and 106.5 months (95% CI, 83.7–129.4 months) for patients with positive F, respectively. There was no significant difference in mean RFS between two groups (p = 0.141). Figure 3**H**. KM curves according to tumor rupture (R). The mean RFS was 216.3 months (95% CI, 198.3–234.2 months) for patients with negative R, and 72.7 months (95% CI, 22.3–123.1 months) for patients with positive R, respectively. There was significant difference in mean RFS between two groups (p = 0.020). Figure 3**I**. KM curves according to caudate involvement (C). The mean RFS was 219.3 months (95% CI, 200.0–238.5 months) for patients with negative C, and 115.6 months (95% CI, 87.0–144.2 months) for patients with positive C, respectively. There was no significant difference in mean EFS between two groups (p = 0.085). Figure 3**J**. KM curves according to lymph node involvement (N). The mean RFS was 209.5 months (95% CI, 189.9–229.1 months) for patients with negative N. The mean RFS of patients with positive N was not estimated because no event had been observed in this group. There was no significant difference in mean EFS between two groups (p = 0.634). Figure 3**K**. KM curves according to distant metastasis (M). The mean RFS was 215.8 months (95% CI, 195.7–235.8 months) for patients with negative M, and 120.6 months (95% CI, 93.5–147.7 months) for patients with positive M, respectively. There was no significant difference in mean RFS between two groups (p = 0.313). Figure 3**L**. KM curves of recurrence-free survival (RFS) according to presence of one or more of VPEFR. The mean RFS was 217.8 months (95% CI, 194.1–241.5 months) for patients with negative VPEFR, and 126.0 months (95% CI, 107.6–144.5 months) for patients with positive VPEFR, respectively. There was no significant difference in mean RFS between two groups (p = 0.366).

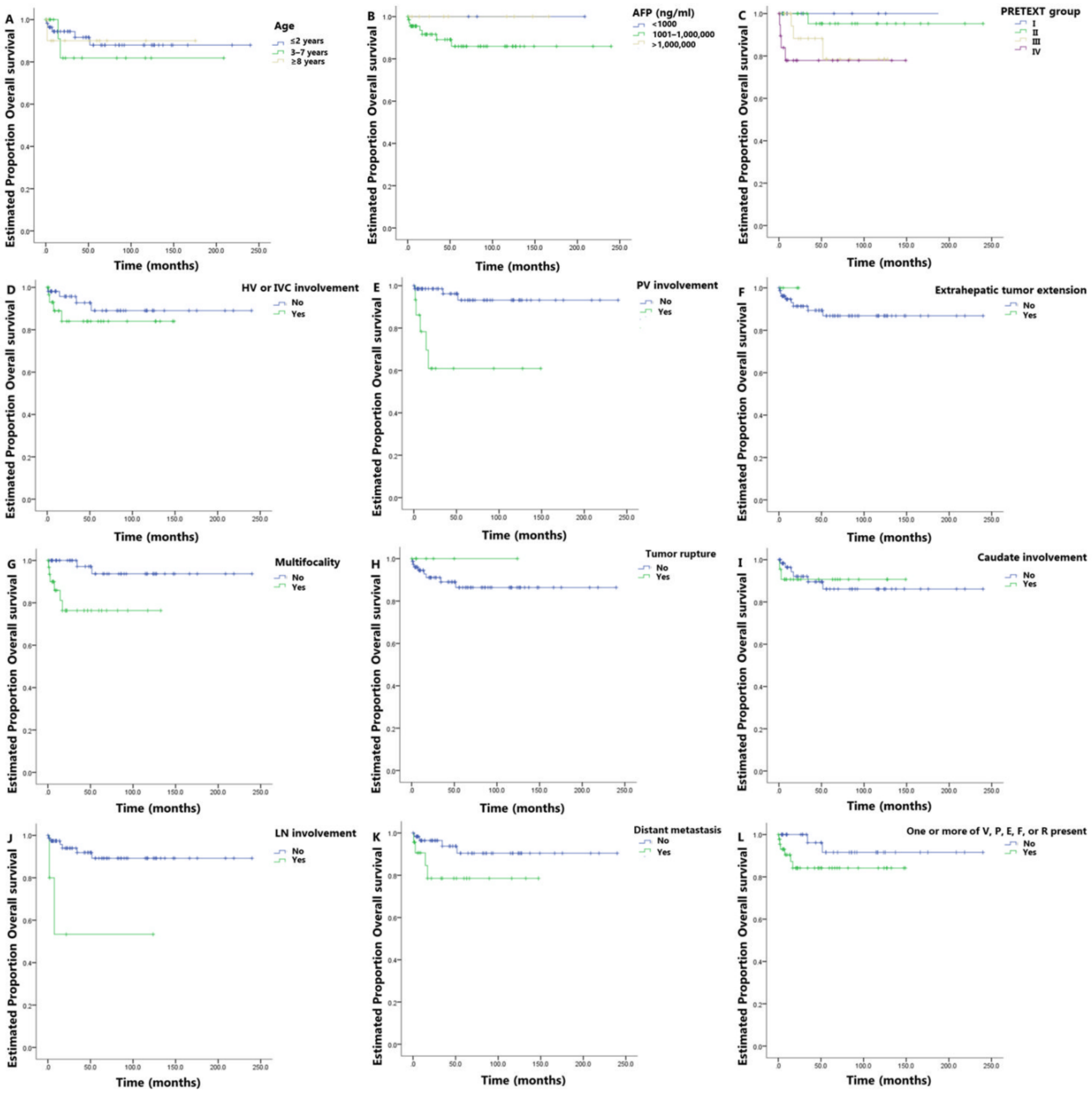

Figure S4. Kaplan-Meier (KM) Curves of Overall Survival (OS). Figure 4**A**. KM curves according to age at initial presentation. The mean OS was 213.9 months (95% CI of 192.1–235.8 months) for patients ≤ 2 years, 173.6 months (95% CI of 129.6–217.6 months) for patients aged 3–8 years, and 158.1 months for patients ≥ 8 years (95% CI of 125.8–190.4 months), respectively. There was no significant difference in mean OS among three groups (p = 0.835). Figure 4**B**. KM curves according to serum alpha-fetoprotein (AFP) levels at initial presentation. The mean OS of patients with AFP < 1000nng/mL and 1001–1000000ng/mL was not estimated because no event was observed in these groups. The mean OS was 209.3 months (95% CI, 187.7−230.9 months) for patients with AFP of 1001–1000000ng/mL. There was no significant difference in mean RFS between two groups (p = 0.476). Figure 4**C**. KM curves according to PRETEXT group. The mean RFS was 230.0 months (95% CI, 211.3−248.7 months) for patients with group II, 107.0 months (95% CI, 86.0−128.0 months) for patients with group III, and 116.7 months (95% CI, 88.7−144.7 months) for patients with group IV, respectively. The mean RFS of PRETEXT group I was not estimated because no event had been observed in this group. There was no significant difference in mean RFS among four groups (p = 0.077). Figure 4**D**. KM curves according to HV or IVC involvement (V). The mean OS was 216.7 months (95% CI, 195.2−238.2 months) for patients with negative V, and 126.4 months (95% CI, 105.9−146.8 months) for patients with positive V, respectively. There was no significant difference in mean EFS between two groups (p = 0.295). Figure 4**E**. KM curves according to PV involvement (P). The mean OS was 225.8 months (95% CI, 210.2−241.4 months) for patients with negative P, and 94.3 months (95% CI, 56.4−132.2 months) for patients with positive P, respectively. There was a significant difference in mean OS between two groups (p < 0.001). Figure 4**F**. KM curves according to

extrahepatic tumor extension (E). The mean RFS was 210.9 months (95% CI, 191.8–230.0 months) for patients with negative E. The mean OS of patients with positive E was not estimated because no event had been observed in this group. There was no significant difference in mean EFS between two groups (p = 0.621). Figure 4**G**. KM curves according to multifocality (F). The mean OS was 227.4 months (95% CI, 210.7–244.1 months) for patients with negative F, and 103.1 months (95% CI, 82.2–124.0 months) for patients with positive F, respectively. There was a significant difference in mean OS between two groups (p = 006). Figure 4**H**. KM curves according to tumor rupture (R). The mean OS was 209.9 months (95% CI, 190.3–229.6 months) for patients with negative R. The mean OS of patients with positive R was not estimated because no event had been observed in this group. There was no significant difference in mean EFS between two groups (p = 0.468). Figure 4**I**. KM curves according to caudate involvement (C). The mean OS was 210.0 months (95% CI, 187.3–232.6 months) for patients with negative C, and 135.2 months (95% CI, 117.1–153.3 months) for patients with positive C, respectively. There was no significant difference in mean OS between two groups (p = 0.992). Figure 4**J**. KM curves according to lymph node involvement (N). The mean OS was 216.6 months (95% CI, 198.6–234.5 months) for patients with negative N, and 68.3 months (95% CI, 10.8–125.8 months) for patients with positive N. There was a significant difference in mean OS between two groups (p = 0.003). Figure 4**K**. KM curves according to distant metastasis (M). The mean OS was 219.6 months (95% CI, 200.4–238.9 months) for patients with negative M, and 117.7 months (95% CI, 91.8–143.7 months) for patients with positive M, respectively. There was no significant difference in mean RFS between two groups (p = 0.084). Figure 4**L**. KM curves according to presence of one or more of VPEFR. The mean OS was 223.3 months (95% CI, 201.3–245.3 months) for patients with negative VPEFR, and 126.6 months (95% CI, 110.1–143.1 months) for patients with positive VPEFR, respectively. There was no significant difference in mean RFS between two groups (p = 0.137).

Supplementary Figure Legend for Figure 2. Kaplan-Meier (KM) Curves of Event-Free Survival (EFS).

Figure 2A. KM curves according to age at initial presentation. The mean EFS was 197.3 months (95% CI, 172.0−222.6 months) for patients ≤ 2 years, 131.9 months (95% CI of 78.4−185.5 months) for patients aged 3−7 years, and 102.0 months for patients ≥ 8 years (95% CI of 53.3−150.8 months), respectively. There was no significant difference in mean EFS among three groups (p = 0.078).

Figure 2B. KM curves according to serum alpha-fetoprotein (AFP) levels at initial presentation. The mean EFS of patients with AFP < 1000nng/mL was not estimated because no event was observed in this group. The mean EFS was 175.9 months (95% CI, 149.8−202.0 months) for patients with AFP of 1001–1000000ng/mL, and 146.3 months (95% CI, 109.8−182.8 months) for patients with AFP more than 1000000 ng/m, respectively. There was no significant difference in mean EFS between two groups (p = 0.389).

Figure 2C. KM curves according to PRETEXT group. The mean EFS was 190.7 months (95% CI, 155.9–222.5 months) for patients with group II, 93.7 months (95% CI, 70.7–116.6 months) for patients with group III, and 92.7 months (95% CI, 60.0−125.5 months) for patients with group IV, respectively. The mean EFS of PRETEXT group I was not estimated because no event had been observed in this group. There was no significant difference in mean EFS among four groups (p = 0.106).

Figure 2D. KM curves according to HV or IVC involvement (V). The mean EFS was 194.6 months (95% CI, 167.9−221.2 months) for patients with negative V, and 97.6 months (95% CI, 72.0−123.3 months) for patients with positive V, respectively. There was no significant difference in mean EFS between two groups (p = 0.079).

Figure 2E. KM curves according to PV involvement (P). The mean EFS was 191.0 months (95% CI, 167.4–214.7 months) for patients with negative P, and 79.9 months (95% CI, 38.5–121.3 months) for patients with positive P, respectively. There was a significant difference in mean EFS between two groups (p = 0.030).

Figure 2F. KM curves according to extrahepatic tumor extension (E). The mean EFS was 179.3 months (95% CI, 155.8–202.8 months) for patients with negative E. The mean EFS of patients with positive E was not estimated because no event had been observed in this group. There was no significant difference in mean EFS between two groups (p = 0.456).

Figure 2G. KM curves according to multifocality (F). The mean EFS was 204.9 months (95% CI, 181.1–228.7 months) for patients with negative F, and 78.1 months (95% CI, 54.4–101.7 months) for patients with positive F, respectively. There was a significant difference in mean EFS between two groups ($p = 0.002$).

Figure 2H. KM curves according to tumor rupture (R). The mean EFS was 186.6 months (95% CI, 163.5–209.7 months) for patients with negative R, and 59.2 months (95% CI, 12.5–105.9 months) for patients with positive R, respectively. There was no significant difference in mean EFS between two groups ($p = 0.089$).

Figure 2I. KM curves according to caudate involvement (C). The mean EFS was 184.9 months (95% CI, 158.6–211.1 months) for patients with negative C, and 105.2 months (95% CI, 75.7–134.6 months) for patients with positive C, respectively. There was no significant difference in mean EFS between two groups ($p = 0.492$).

Figure 2J. KM curves according to lymph node involvement (N). The mean EFS was 183.8 months (95% CI, 160.5–207.1 months) for patients with negative N, and 67.9 months (95% CI, 9.9–125.8 months) for patients with positive N, respectively. There was no significant difference in mean EFS between two groups ($p = 0.133$).

Figure 2K. KM curves according to distant metastasis (M). The mean EFS was 199.7 months (95% CI, 175.8–223.7 months) for patients with negative M, and 83.2 months (95% CI, 53.8–112.7 months) for patients with positive M, respectively. There was a significant difference in mean EFS between two groups ($p = 0.003$).

Figure 2L. KM curves according to presence of one or more of VPEFR. The mean EFS was 204.8 months (95% CI, 176.6–233.0 months) for patients with negative VPEFR, and 100.6 months (95% CI, 79.7–121.4 months) for patients with positive VPEFR, respectively. There was a significant difference in mean EFS between two groups ($p = 0.035$).

© 2019 by the authors. Licensee MDPI, Basel, Switzerland. This article is an open access article distributed under the terms and conditions of the Creative Commons Attribution (CC BY) license (http://creativecommons.org/licenses/by/4.0/).
